# Predicting asthma in preschool children with asthma symptoms: study rationale and design

**DOI:** 10.1186/1471-2466-12-65

**Published:** 2012-10-16

**Authors:** Esther Hafkamp-de Groen, Hester F Lingsma, Daan Caudri, Alet Wijga, Vincent WV Jaddoe, Ewout W Steyerberg, Johan C de Jongste, Hein Raat

**Affiliations:** 1 The Generation R Study Group, Erasmus MC, Rotterdam, The Netherlands; 2 Department of Public Health, Erasmus MC, Rotterdam, The Netherlands; 3 Department of Paediatrics, Division of Respiratory Medicine, Erasmus MC-Sophia Children’s Hospital, Rotterdam, The Netherlands; 4 Centre for Prevention and Health Services Research / National Institute for Public Health and the Environment, Bilthoven, The Netherlands; 5 Department of Epidemiology, Erasmus MC, Rotterdam, The Netherlands; 6 Department of Paediatrics, Erasmus MC-Sophia Children’s Hospital, Rotterdam, The Netherlands

**Keywords:** Asthma Risk Appraisal Tool, Children, External validation, Prediction, Well-child care

## Abstract

**Background:**

In well-child care it is difficult to determine whether preschool children with asthma symptoms actually have or will develop asthma at school age. The PIAMA (Prevention and Incidence of Asthma and Mite Allergy) Risk Score has been proposed as an instrument that predicts asthma at school age, using eight easy obtainable parameters, assessed at the time of first asthma symptoms at preschool age. The aim of this study is to present the rationale and design of a study 1) to externally validate and update the PIAMA Risk Score, 2) to develop an Asthma Risk Appraisal Tool to predict asthma at school age in (specific subgroups of) preschool children with asthma symptoms and 3) to test implementation of the Asthma Risk Appraisal Tool in well-child care.

**Methods and design:**

The study will be performed within the framework of Generation R, a prospective multi-ethnic cohort study. In total, consent for postnatal follow-up was obtained from 7893 children, born between 2002 and 2006. At preschool age the PIAMA Risk Score will be assessed and used to predict asthma at school age. Discrimination (C-index) and calibration will be assessed for the external validation. We will study whether the predictive ability of the PIAMA Risk Score can be improved by removing or adding predictors (e.g. preterm birth). The (updated) PIAMA Risk Score will be converted to the Asthma Risk Appraisal Tool- to predict asthma at school age in preschool children with asthma symptoms. Additionally, we will conduct a pilot study to test implementation of the Asthma Risk Appraisal Tool in well-child care.

**Discussion:**

Application of the Asthma Risk Appraisal Tool in well-child care will help to distinguish preschool children at high- and low-risk of developing asthma at school age when asthma symptoms appear.

This study will increase knowledge about the validity of the PIAMA risk score and might improve risk assessment of developing asthma at school age in (specific subgroups of) preschool children, who present with asthma symptoms at well-child care.

## Background

Asthma symptoms in preschool children are non-specific. It is therefore difficult to determine which preschool children with asthma symptoms actually have or will develop asthma at school age [1]. A recent study has shown that both undertreatment and overtreatment of asthma in children between ages 2 and 8 years seem common [2]. Inadequate risk assessment of asthma when children present with asthma symptoms at well-child care may be an important cause of inadequate treatment of childhood asthma. To improve early diagnosis and management of asthma symptoms, we reasoned that early detection of preschool children at high risk of developing asthma at school age is important.

In this study we present the rationale and design of a study focusing on risk assessment of asthma in well-child care. Well-child care physicians and nurses have routine contact with about 90% of all preschool children and their families [3] and therefore can play an important role in 1) early detection of children with asthma symptoms in the general population, 2) risk assessment of asthma in early detected children and 3) adequate monitoring and counselling of children at high risk of asthma. The first and third step are currently being studied in a randomised controlled trial ‘to evaluate the effectivity of early detection and counselling of preschool asthma symptoms within well-child care’ [4]. However, the second step of asthma risk assessment in children who are detected early in life is not yet available within well-child care. There is a need for an Asthma Risk Appraisal Tool to support well-child care professionals when a preschool child presents with asthma symptoms.

To estimate the risk of developing asthma at school age at the time children have asthma symptoms in preschool years, a risk score (i.e. prediction model) may be a suitable tool. A tool like this could support the communication between well-child care professionals and parents of children at risk of developing asthma. Several studies previously developed a prediction model for asthma [5-12]. It is complicated to compare these studies, because definitions and age of asthma differed. Many studies used information up to a fixed age, irrespective of the age of symptom onset [6,8,10,11]. The PIAMA (Prevention and Incidence of Asthma and Mite Allergy) Risk Score has been proposed as an instrument that predicts asthma at age 7–8 years, using eight easy obtainable parameters, assessed at the time of first asthma symptoms at preschool age [7]. The PIAMA Risk Score discriminated between asthmatic and non-asthmatic children (internally validated area under the curve, AUC = 0.72) and may be a suitable tool for use in well-child care. Prediction models are mathematical models based on available patient data from a certain setting. Before use of a prediction model can be recommended in practice, external validation is mandatory to determine the ability of a model to reliably predict the outcome in other populations and settings [7].

The main objective is to present the rationale and design of a study to externally validate and update the PIAMA Risk Score. Furthermore, an Asthma Risk Appraisal Tool will be developed to predict asthma at school age in (specific subgroups of) preschool children with asthma symptoms. We will conduct a pilot test of the Asthma Risk Appraisal Tool within well-child care.

By describing the rationale and design of our study we give insight into the framework of our study. This framework concerns the process of external validation and updating of a prediction rule, development of an application tool and assessment of whether the tool can be implemented into practice. This study will help others to convert prediction rules into practice.

## Methods and design

### Design and setting

Our study will be embedded in Generation R, a prospective population-based, multi-ethnic cohort study. In total, consent for postnatal follow-up was obtained from 7893 children, born between April 2002 and January 2006 [13]. Questionnaires for parental completion, partly based on the International Study of Asthma and Allergies in Childhood (ISAAC) core questionnaires [14], were sent to the parents during pregnancy and when the children were aged 1, 2, 3, 4 and 6 years (N=7893) [15]. Response rates for these questionnaires were 71%, 76%, 72%, 73% and 68% respectively. Data collection at child’s age of 9 years is currently ongoing. In this study, children will be included if at least one positive response was given to the following questions in the annual questionnaires at age 1 to 4 years: “Has your child had wheezing in the last 12 months?” and “Has your child had cough during the night, when he/she did not have a cold or a chest infection, in the last 12 months?” The present study was conducted in accordance with the guidelines proposed in the Declaration of Helsinki, and is approved by the Medical Ethical Committee of the Erasmus Medical Centre (MEC 217.595/2002/202). Written consent was obtained from all participating parents.

### Asthma outcomes

The outcome that is predicted with the PIAMA Risk Score is asthma at school age. In the development study (PIAMA) the following 3 items were used for the case definition of asthma: (1) at least 1 episode of wheezing in the last 12 months; (2) inhaled steroids prescribed by a medical doctor in the last 12 months; and (3) a doctor’s diagnosis of asthma (a parental report of a doctor’s diagnosis of asthma at any time and a parental report of asthma in the last 12 months). In the analyses children were only considered positive for asthma if they had 1 or more positive items at age 7 years and 1 or more positive items at age 8 years [7].

Within the validation data (Generation R), we aim to use the same asthma definition as used in PIAMA and we aim to select only children with ‘active asthma’ or clinically relevant chronic asthma symptoms. First, we will define asthma at the age of 6 years in the children who have ever had reported asthma symptoms before the age of 4 years. Additionally, the analyses will be repeated in children at age 9 years. At age 9 years spirometry will be performed in children at the research center. Spirometry is used to improve the accuracy of an asthma diagnoses and will be enable us to compare asthma outcomes based on parental reports to asthma outcomes based on spirometry.

### Preschool predictors

The eight predictor variables used in the PIAMA Risk Score are: 1) sex, 2) post-term delivery, 3) parental education, 4) parental inhalation medication, 5) child’s wheezing frequency, 6) wheezing/dyspnea apart from colds, 7) serious infections and 8) doctor’s diagnosis of eczema and eczematous rash present. The variables wheezing/dyspnea apart from colds and parental inhalation medication are not available within the Generation R Study at preschool age. For parental inhalation medication a proxy variable of parental asthma is available. Information on sex and pregnancy duration are obtained from medical records; parental education and asthma are established using questionnaires during pregnancy; wheezing frequency, respiratory tract infections and eczema are measured using questionnaires at the ages of 1, 2, 3 and 4 years.

### External validation

As a first step we will compare the distribution of the predictors of the PIAMA Risk Score and the asthma outcome in the development (PIAMA) and validation (Generation R) data to determine whether the datasets are comparable. Univariate logistic regression analyses will be performed to establish the effect of the different predictors on asthma at the age of 6 and 9 years. The resulting univariate odds ratios (ORs) will be compared with ORs in the development sample as reported for the PIAMA model. Next, the multivariate PIAMA model will be fitted in the validation sample to compare the multivariate ORs. Finally we will calculate the predicted probability to develop asthma for each child in the validation sample, based on the PIAMA score. These predicted probabilities are used to assess the external validity of the PIAMA model, in terms of calibration and discrimination. Calibration refers to the agreement between observed and predicted outcomes. The extent of over- or underestimation relative to the observed and predicted rate will be explored graphically using validation plots. We will assess calibration-in-the-large by fitting a logistic regression model with the model predictions as an offset variable. The intercept indicates whether predictions are systematically too low or too high, and should ideally be zero. The calibration slope reflects the average effects of the predictors in the model and will be estimated in a logistic regression model with the logit of the model predictions as the only predictor. For a perfect model, the slope is equal to 1. The Concordance-index (C-index) or Area Under the receiver operating characteristic Curve (AUC) and 95% confidence interval (CI) will be used to assess the ability of the model to discriminate children with and without asthma. The external validation will also be performed in specific subgroups (e.g. at different ages or in ethnic and socioeconomic subgroups of preschool children, see subgroup analysis). To interpret any differences in C-indices, we will consider benchmark values as recently proposed [16].

### Updating

After external validation we will assess whether the predictive performance of the PIAMA model remains stable or improves by deleting or adding predictors that are available in the validation data.

By removing predictors, a more simple risk score will be created. The predictive performance of such a simple risk score will be compared with the predictive performance of the PIAMA Risk Score. A simpler risk score is preferable for application in practice [17]. Potential additional predictors include e.g. child’s ethnicity, preterm birth, sleeping problems due to asthma symptoms, doctor visits due to asthma symptoms, wheezing patterns, allergy or general health. To study the prognostic value of additional predictors, we will refit the PIAMA Risk Score in the validation data and consequently add the new predictors. We will calculate the increase in AUC with 95% CI, and the p-value from the likelihood ratio test for improvement of goodness of fit. This will result in an updated PIAMA Risk Score. For optimal precision of the estimated coefficients, the updated PIAMA Risk Score will be fitted to the combined PIAMA and Generation R data.

### Subgroup analysis

The PIAMA Risk Score was developed within a general population. However, it is known that children of ethnic minorities and children with low socioeconomic status are at high risk of developing asthma. Within well-child care it is important to give attention to high risk groups. Therefore, it is important to test the predictive ability of the PIAMA Risk Score in both the general population and in specific subgroups (e.g. at different ages or in children of ethnic minorities and children with low socioeconomic status).

### Development of asthma risk appraisal tool

We will convert the updated PIAMA Risk Score to a computer-assisted tool, the so called ‘Asthma Risk Appraisal Tool’. The best cut-off scores of the Asthma Risk Appraisal Tool will be studied within the validation study. In an expert meeting we will discuss which decisions will follow the cut-offs: referral to general practitioner (=indirect referral to pediatrician)/ asthma nurse, extra consultation moment at well-child care, personal advise/counselling. The aim is to create an easy applicable (computer-assisted) tool for use of the PIAMA Risk Score in well-child care. A previous study developed a similar risk assessment tool to early detect children with global developmental disabilities in well-child care [18]. A computer-assisted risk assessment tool heightens the uniformity of practice.

### Pilot testing

After development of the Asthma Risk Appraisal Tool, the tool will be tested in a pilot study within well-child care. The pilot test will be conducted in the Rotterdam-Rijnmond area that contains both rural and metropolitan and ethnically diverse sub-regions. The implementation will involve 3 varied well-child care teams (including one or more well-child care physicians, nurses and medical assistants per team that provide services to a certain group of preschool children in a distinct geographical region). It is aimed to pilot the Asthma Risk Appraisal Tool to 100 children/families. So, a total of 300 children/families are aimed to be included in the pilot study.

When children present with asthma symptoms, the Asthma Risk Appraisal Tool will be applied. In the pilot study we will assess how many preschool children were detected as high-risk of developing asthma at the ages of 6 and 9 years by the Asthma Risk Appraisal Tool. We will evaluate which decisions cq. actions were taken by physicians/nurses after the use of the Asthma Risk Appraisal Tool (and how many times). Evaluation of the effectivity of implementation of the Asthma Risk Appraisal Tool in well-child care practice is outside the framework of this study.

### Sample size

At least 100 patients with the outcome and 100 without the outcome are needed for reliable external validation of a prediction model [19].

Sample size at the age of 6 years: Assuming a prevalence of asthma of 5% at the age of 6 years (and it is known that 3967 children have ever had asthma symptoms at the age of 4 years) the Generation R study will have approximately 198 children aged 6 years [19]. This implies that our effective sample size at the age of 6 years is sufficiently large for the primary aim of this study.

## Discussion

We present the rationale and design of a study to externally validate and update the PIAMA Risk Score and to develop and test an application of the PIAMA Risk Score to predict asthma at school age in (specific subgroups of) preschool children with asthma symptoms. This Asthma Risk Appraisal Tool might be used in well-child care as an Asthma Risk Appraisal Tool in preschool children already detected with asthma symptoms.

Several studies previously developed a prediction model to predict asthma [5-12]. It is complicated to compare these studies, because definitions and age of asthma differs and it is unknown which definition of asthma truly identifies the disease. Many studies used information up to a fixed age irrespective of the age of symptom onset [6,8,10,11]. Some of the prediction models included blood tests [6,11,12]. Prediction models including blood tests are not feasible in well-child care, given the (very) low acceptance of drawing blood in the setting of prevention by parents and children, the lack of funding for laboratory tests in preventive healthcare, and because laboratory results should be awaited. Therefore, the PIAMA Risk Score - including only easy obtainable parameters - is preferred above prediction models including blood tests. The PIAMA Risk Score has been compared to the asthma predictive index developed by Castro-Rodriquez et al. [6] and showed a better predictive ability, and also performed better than a doctors diagnosis of asthma at the same age [7].

This study benefits from a longitudinal design, which enables us to collect repeated measurements of predictors at preschool age. In this way we can identify the age at onset of first symptoms, unlike some earlier studies who predict asthma at fixed ages [6,8,10,11]. The ages at which asthma symptoms appear most frequently is the time that children will regularly visit well-child care and when prediction of asthma becomes relevant. Furthermore, there will be little differences in design and analysis between the development and the validation study. It is our intention to develop an Asthma Risk Appraisal Tool integrated in well-child care at preschool age in such a way that it has maximal opportunity for future wide-spread implementation, once proved useful.

There are several reasons why early detection followed by risk assessment of asthma is important: early detection of preschool children at high risk of developing asthma at school age will contribute to adequate and early management, resulting in fewer asthma symptoms, while improving child’s quality of life [4,20]. Furthermore, for parents of preschool children it is important to know the risk of developing asthma at school age, and the options for treatment or intervention to reduce or prevent progression of asthma symptoms.

Risk assessment is important because in well-child care (in the Netherlands) task reallocation is ongoing: an approach where children and families with the highest risks on health and psychosocial problems receive higher levels of preventive care and monitoring. Those with low risk of health and psychosocial problems should be offered care at a basic level in terms of frequency, content and type of professional. The background of this approach is often budgetary pressure. In most cases nowadays, risk selection is carried out by a trained healthcare assistant based on predefined factors at preschool age (e.g. socioeconomic status, single parenting, child health, paternal psychopathology). Although child’s health at preschool age is one of the factors which is included in the approach of risk selection at school age, no specific attention is given to preschool child’s asthma symptoms or preschool child’s risk of developing asthma at school age. To prevent inadequate treatment of childhood asthma and to prevent that children with an increased risk of asthma are lost to follow up by primary and secondary healthcare, it is important to assess the risk of developing asthma at school age when preschool children present with asthma symptoms at well-child care.

The aim of improved risk assessment of asthma is to achieve optimal asthma management without delay in preschool children with symptoms suggestive of asthma who are at high risk of developing asthma. In turn, the aim of optimal asthma management is to reduce and prevent the burden of asthma in the future and to improve the child’s quality of life. However, this topic is outside the framework of this study. After pilot testing and implementation, a randomised controlled trial is a possible next step to evaluate the effectivity of the use of an Asthma Risk Appraisal Tool in well-child care to support professionals in risk assessment of asthma, when preschool children present with asthma symptoms.

## Conclusion

This study will increase knowledge about the external validity of the PIAMA risk score and might improve risk assessment of developing asthma at school age in (specific subgroups of) preschool children, who present with asthma symptoms at well-child care.

## Abbreviations

C-index: Concordance-index; ISAAC: International study of asthma and allergies in childhood; ORs: Odds ratios; PIAMA: Prevention and incidence of asthma and mite allergy.

## Competing interests

All authors declare that they have no competing interests.

## Authors’ contributions

The study was initiated by EH and HR. HL, DC, AW, VWV, JCJ, ES and HR participated in the study concept and design. All authors reviewed and approved the final version of this manuscript.

## Pre-publication history

The pre-publication history for this paper can be accessed here:

http://www.biomedcentral.com/1471-2466/12/65/prepub
